# Can pathoanatomical pathways of degeneration in lumbar motion segments be identified by clustering MRI findings

**DOI:** 10.1186/1471-2474-14-198

**Published:** 2013-07-01

**Authors:** Rikke K Jensen, Tue S Jensen, Per Kjaer, Peter Kent

**Affiliations:** 1 Research Department, Spine Centre of Southern Denmark, Hospital Lillebaelt, Middelfart, Denmark; 2 Institute of Regional Health Services Research, University of Southern Denmark, Middelfart, Denmark; 3 Institute of Sports Science and Clinical Biomechanics, University of Southern Denmark, Odense, Denmark

**Keywords:** Low back pain, Disc degeneration, Magnetic Resonance Imaging, Subgroup, Latent Class Analysis

## Abstract

**Background:**

Magnetic Resonance Imaging (MRI) is the gold standard for detailed visualisation of spinal pathological and degenerative processes, but the prevailing view is that such imaging findings have little or no clinical relevance for low back pain. This is because these findings appear to have little association with treatment effects in clinical populations, and mostly a weak association with the presence of pain in the general population.

However, almost all research into these associations is based on the examination of individual MRI findings, despite its being very common for multiple MRI findings to coexist. Therefore, this proof-of-concept study investigated the capacity of a multivariable statistical method to identify clusters of MRI findings and for those clusters to be grouped into pathways of vertebral degeneration.

**Methods:**

This study is a secondary analysis of data from 631 patients, from an outpatient spine clinic, who had been screened for inclusion in a randomised controlled trial. The available data created a total sample pool of 3,155 vertebral motion segments. The mean age of the cohort was 42 years (SD 10.8, range 18–73) and 54% were women.

MRI images were quantitatively coded by an experienced musculoskeletal research radiologist using a detailed and standardised research MRI evaluation protocol that has demonstrated high reproducibility. Comprehensive MRI findings descriptive of the disco-vertebral component of lumbar vertebrae were clustered using Latent Class Analysis. Two pairs of researchers, each containing an experienced MRI researcher, then independently categorised the clusters into hypothetical pathoanatomic pathways based on the known histological changes of discovertebral degeneration.

**Results:**

Twelve clusters of MRI findings were identified, described and grouped into five different hypothetical pathways of degeneration that appear to have face validity.

**Conclusions:**

This study has shown that Latent Class Analysis can be used to identify clusters of MRI findings from people with LBP and that those clusters can be grouped into degenerative pathways that are biologically plausible. If these clusters of MRI findings are reproducible in other datasets of similar patients, they may form a stable platform to investigate the relationship between degenerative pathways and clinically important characteristics such as pain and activity limitation.

## Background

While it is widely accepted that low back pain (LBP) is a bio-psycho-social condition, concern has recently been expressed that LBP research is moving too far from the biological component [1]. However, despite the large body of previous research attempting to identify specific biological reasons for LBP, there are still more questions than answers. The clinical consequence of this uncertainty is that only approximately 15% of primary care patients with LBP are classified with a specific pathoanatomic diagnosis, with the remaining 85% being diagnosed with non-specific LBP [2].

In research, non-specific LBP is often conceptualised as one condition, although most clinicians and researchers believe that non-specific LBP is actually a heterogenous mix of conditions [3]. This distinction occurs because, although many methods to subgroup non-specific LBP have been proposed, the evidence for the validity of almost all these approaches remains very tentative [4]. It is also the case that almost all subgrouping methods that have demonstrated any treatment validity are not based on pathoanatomy [5], the exception being the established association between pain centralisation and discogenic pain [6,7].

One way of categorising patients into diagnostic subgroups is by pathoanatomy identified with Magnetic Resonance Imaging (MRI). MRI has increasingly replaced other imaging modalities in the diagnosis of LBP because of the level of detail it provides. Paradoxically, despite the unprecedented level of detail available on spinal pathological or degenerative findings, the prevailing view is that they have little clinical relevance [8-10]. This is because these findings seem to have little or no association with treatment effect [11] in clinical populations, and mostly a weak and inconsistent association with the presence of pain in the general population [12]. Furthermore, when using MRI, there is normally no way to distinguish age-related degeneration (believed to be non-painful) and ‘pathological’ or trauma-related degeneration (believed to be painful). Similarly, it has been reported that normal aging of the motion segment and pathological degeneration cannot be distinguished at a histological level [13], suggesting that the biological process is the same in normal aging but is accelerated in the pathological state. Therefore, the biological age of a vertebral motion segment is not necessarily the same as the actual chronological age of the person.

However, investigation into associations between MRI findings and pain are complicated by numerous MRI findings being present at the same time. For example, vertebral endplate signal changes and vertebral disc herniations almost always co-exist with other degenerative disc findings, such as reduction of height and reduction of the signal intensity of the disc [14-17]. Despite this, most previous research has focused solely on the association between single imaging findings and pain or other clinical outcomes. It is only recently that researchers have started to explore the potential for answers within this complexity, for example Cheung and co-workers[18], who have reported a positive correlation between the sum of degenerative disc MRI findings and low back pain.

Classification systems exist for categorising the detailed information on the physiological condition of a motion segment that MRI provides. For example, the Pfirrmann classification [19] is a descriptive tool for classifying intervertebral disc degeneration. However, such classification systems also typically focus on findings related to one or two anatomic entities, such as the height and signal intensity of the intervertebral disc, leaving un-described other relevant entities such as vertebral endplate irregularity, vertebral endplate signal changes and disc herniations.

It would be useful if there were methods to better model the multivariable relationships between clusters of MRI findings, as these might provide a clearer understanding of how pathoanatomical processes are expressed across all the structures associated with a vertebral segment. Furthermore, the identification of such processes, and an ability of such clusters to stage the biological age of a vertebral segment, might allow better insight into the relationship between degenerative processes and clinical characteristics such as pain and activity limitation.

Therefore, the aims of this study were to investigate: (i) how multiple MRI findings from lumbar spine motion segments cluster together in patients with chronic LBP, and (ii) to classify these clusters of MRI findings into hypothetical pathoanatomical pathways.

## Methods

This was a ‘proof of concept’ study designed to test a novel analytical method and set hypotheses about pathoanatomical pathways of vertebral segment degeneration. It used cross-sectional data and a probabilistic (Bayesian) Latent Class Analysis approach to the multivariable clustering of MRI findings.

### Study sample

This study is a secondary analysis of data from a cohort of patients who were potential participants in a randomised controlled trial [20]. All participants attended the same publically-funded outpatient spine clinic (The Spine Centre of Southern Denmark) where they had been referred from the primary care sector for a multidisciplinary evaluation. In this clinical setting, from June 2006 to June 2008, MRI was routinely performed on all patients (who had no contraindications for MRI) who met the following criteria: (a) LBP or leg pain of at least 3 on an 11-point Numerical Rating Scale, (b) duration of current symptoms from 2 to 12 months, and (c) age above 18 years.

### Data collection

MRI was performed with a 0.2 T MRI-system (Magnetom Open Viva; Siemens AG, Erlangen, Germany). A body spine surface coil was used for imaging of the lumbar region, with the study subjects in the supine position. The imaging protocol consisted of the following sequences:

• Localizer sequence, 40/10/40 (TR/TE/flip angle), two coronal and three sagittal images in orthogonal planes

• Sagittal T1-weighted spin echo, 621/26 (TR/TE), 144 x 256 matrix, 300 mm FOV, and 11 slices 4 mm wide, distance factor 0.20.

• Sagittal T2-weighted turbo spin echo, 4609/134 (TR/effective TE), 210 x 256 matrix, 300 mm FOV, and 11 slices 4 mm wide, distance factor 0.20.

• Axial T1-weighted spin echo, 720/26 (TR/TE), 192 x 256 matrix, 240 mm FOV, and 15 slices 5 mm wide, distance factor 0.25.

• Axial T2-weighted turbo spin echo, 6415/134 (TR/effective TE), 180 x 256 matrix, 250 mm FOV, and 15 slices 5 mm wide, distance factor 0.25.

• Axial images were performed on the three lower lumbar levels. If herniations were present at higher lumbar levels, relevant supplementing axial series were performed.

The images were quantitatively coded using a detailed and standardised research MRI evaluation protocol [21,22]. The evaluation was performed by an experienced musculoskeletal research radiologist who was blinded to any participant information other than name, age and sex.

### Variables of interest

The following MRI variables were included in the current study: intervertebral disc height, disc signal intensity, disc herniations, size and type of vertebral endplate signal changes (VESC), irregularity of the vertebral endplate, osteophytes and spondylolisthesis. Each MRI variable was coded on two to seven different pathoanatomical stages (categories), as detailed in Additional file 1.

Previous testing of this MRI evaluation protocol when used by the same radiologist has shown substantial to almost perfect inter- and intra-observer reliability for the VESC variables (Kappa ranging from 0.73 to 1.0), and moderate to substantial reliability for other endplate related findings (Kappa ranging from 0.52 to 0.72) [21]. Evaluation of intervertebral disc-related changes has shown moderate to almost perfect reliability (Kappa ranging from 0.59 to 0.97) [22]. Anterolisthesis was evaluated according to Meyerding classification system [23].

### Statistical analyses

MRI findings were clustered using the multivariable statistical program ‘SNOB’ (Monash Data Mining Centre, Monash University, Melbourne, Victoria, Australia), which is a form of Latent Class Analysis. SNOB uses probabilistic mixture modelling and the minimum message length principle to determine the optimum class structure (clusters) within the data. Minimum message length is an automated statistical method to determine which of the possible models of class structure and explained variance are the most parsimonious and explanatory. Latent Class Analysis has a number of advantages over traditional cluster analysis techniques, including: greater classification accuracy, the ability to manage variables of all data types (dichotomous, ordinal and continuous), and a tolerance of missing data [24-26].

Analysis occurred at the level of individual vertebral segments, as lumbar segments within an individual person may each have a different ‘pathoanatomical age’. Therefore, every participating person contributed five lumbar vertebral segments to the analysis.

The Latent Class Analysis identified the optimal number of clusters and also the cluster membership for each vertebral segment. We used this information to determine the proportion of vertebral segments within each cluster that displayed each coding category on each MRI variable and the proportion of vertebral levels (L1/2, L2/3, L3/4 L4/5 and L5/S1) within each cluster. These data were calculated and graphed using Excel 2008 for Mac version 12.2.8 (Microsoft Corporation, Redmond, WA, USA).

Then two pairs of researchers, each containing an experienced MRI researcher, independently categorised the clusters into hypothetical pathoanatomic pathways based on the face validity of known histological changes of discovertebral degeneration. These histological changes had been identified by an (unpublished) electronic literature search (PubMed and Medline) and review. The pathways categorised by the pairs of researchers were compared and any differences resolved by discussion.

A post-hoc calculation of the mean (SD) chronological age of the motion segments within each cluster was also performed. This metric was used to test if the chronological age of each cluster challenged or supported the concept of ‘pathoanatomical age’ inherent in the pathways.

### Ethics

This analysis was based on existing data collected for a randomised controlled trial[20] approved by the Ethics Committee for the Region of Southern Denmark (approval # S-VF-20060111), registered in ClinicalTrials.gov (Identifier # NCT00454792) and performed following the Declaration of Helsinki principles.

## Results

### Population

MRI findings and basic demographic data were available on 631 patients who had been screened for inclusion in the original studies. All were included in the current study cohort, which created a total sample pool of 3,155 vertebral motion segments. The mean age of the cohort was 42 years (SD 10.8, range 18–73) and 54% were women. Detailed descriptive data of the MRI findings at a whole group level are tabulated in Additional file 1.

### Clusters of MRI findings

**Figure 1:**
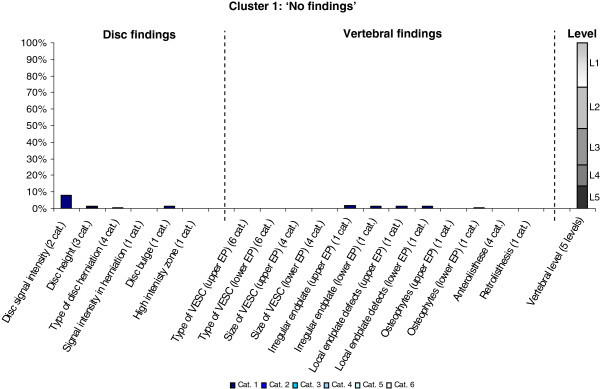
**Prevalence of MRI findings within Cluster 1.** This cluster contained 51.6% of the 3155 vertebral motion-segments. **(a)** The vertical bars on the graph represent the proportion of vertebral motion segments present within each coding category for each MRI finding. The bars on the graph do not show the ‘category 0’ as that represents the presence of only normal findings. For pathologies with only one category it represents the presence of the pathology. For ‘disc signal intensity’, ‘disc height’ and ‘size of VESC’ the categories refer to increasing severity or size of pathology with ‘Category 1’ being the least severe. For ‘herniations’, the categories represent the following types: 1 = focal protrusion; 2 = broad-based protrusion; 3 = extrusion; 4 = sequestration. For ‘type of VESC’, the categories refer to the following types: 1 = Type I; 2 = Type II; 3 = Type III; 4 = mixed Type I/II; 5 = mixed Type II/III; 6 = mixed Type I/III. For ‘anterolisthesis’, the categories refer to the Meyerdings classification. Detailed information on proportions of categories of MRI findings is described in Additional file 2. **(b)** The mean age of the motion segments was 39 years (SD 10) and the proportion of women was 58%. **(c)** VESC = vertebral endplate signal change; Cat. = coding categories; EP = endplate.

**Figure 2:**
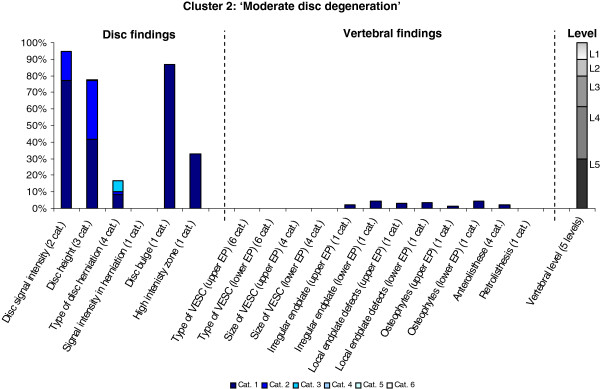
**Proportion of categories of MRI findings for Cluster 2 containing 14.9% of the 3155 motion-segments. (a)** The vertical bars on the graph represent the proportion of vertebral motion segments present within each coding category for each MRI finding. The bars on the graph do not show the ‘Category 0’, as that represents the presence of only normal findings. For pathologies with only one category, it represents the presence of the pathology. For ‘disc signal intensity’, ‘disc height’ and ‘size of VESC’ the categories refer to increasing severity or size of pathology with ‘Category 1’ being the least severe. For ‘herniations’, the categories represent the following types: 1 = focal protrusion; 2 = broad-based protrusion; 3 = extrusion; 4 = sequestration. For ‘type of VESC’, the categories refer to the following types: 1 = Type I; 2 = Type II; 3 = Type III; 4 = mixed Type I/II; 5 = mixed Type II/III; 6 = mixed Type I/III. For ‘anterolisthesis’ the categories refer to the Meyerdings classification. Detailed information on proportions of categories of MRI findings is described in Additional file 2. **(b)** The mean age of the motion segments was 46 years (SD 11) and the proportion of women was 55%. **(c)** VESC = vertebral endplate signal change; Cat. = categories; EP = endplate.

**Figure 3:**
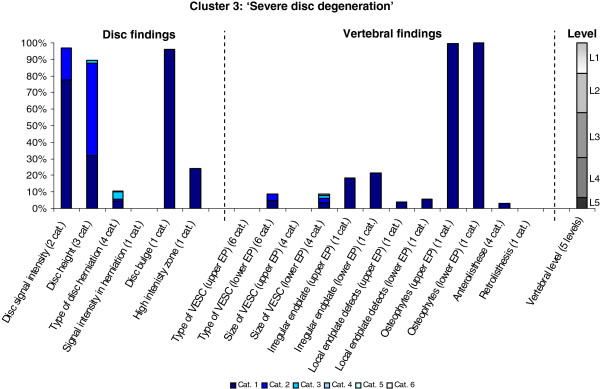
**Proportion of categories of MRI findings for Cluster 3 containing 7.7% of the 3155 motion-segments. (a)** The vertical bars on the graph represent the proportion of vertebral motion segments present within each coding category for each MRI finding. The bars on the graph do not show the ‘Category 0’, as that represents the presence of only normal findings. For pathologies with only one category, it represents the presence of the pathology. For ‘disc signal intensity’, ‘disc height’ and ‘size of VESC’, the categories refer to increasing severity or size of pathology with ‘Category 1’ being the least severe. For ‘herniations’, the categories represent the following types: 1 = focal protrusion; 2 = broad-based protrusion; 3 = extrusion; 4 = sequestration. For ‘type of VESC’, the categories refer to the following types: 1 = Type I; 2 = Type II; 3 = Type III; 4 = mixed Type I/II; 5 = mixed Type II/III; 6 = mixed Type I/III. For ‘anterolisthesis’ the categories refer to the Meyerdings classification. Detailed information on proportions of categories of MRI findings is described in Additional file 2. **(b)** The mean age of the motion segments was 47 years (SD 10) and the proportion of women was 47%. **(c)** VESC = vertebral endplate signal change; Cat. = categories; EP = endplate.

**Figure 4:**
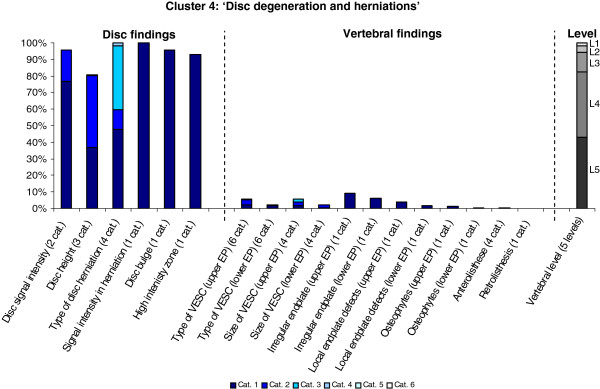
**Proportion of categories of MRI findings for Cluster 4 containing 5.5% of the 3155 motion-segments. (a)** The vertical bars on the graph represent the proportion of vertebral motion segments present within each coding category for each MRI finding. The bars on the graph do not show the ‘Category 0’, as that represents the presence of only normal findings. For pathologies with only one category, it represents the presence of the pathology. For ‘disc signal intensity’, ‘disc height’ and ‘size of VESC’, the categories refer to increasing severity or size of pathology with ‘Category 1’ being the least severe. For ‘herniations’, the categories represent the following types: 1 = focal protrusion; 2 = broad-based protrusion; 3 = extrusion; 4 = sequestration. For ‘type of VESC’ the categories refer to the following types: 1 = Type I; 2 = Type II; 3 = Type III; 4 = mixed Type I/II; 5 = mixed Type II/III; 6 = mixed Type I/III. For ‘anterolisthesis’, the categories refer to the Meyerdings classification. Detailed information on proportions of categories of MRI findings is described in Additional file 2. **(b)** The mean age of the motion segments was 41 years (SD 10) and the proportion of women was 48%. **(c)** VESC = vertebral endplate signal change; Cat. = categories; EP = endplate.

**Figure 5:**
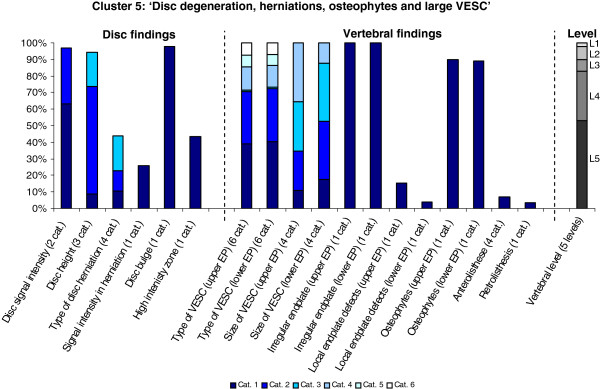
**Proportion of categories of MRI findings for Cluster 5 containing 5.4% of the 3155 motion-segments. (a)** The vertical bars on the graph represent the proportion of vertebral motion segments present within each coding category for each MRI finding. The bars on the graph do not show the ‘Category 0’, as that represents the presence of only normal findings. For pathologies with only one category, it represents the presence of the pathology. For ‘disc signal intensity’, ‘disc height’ and ‘size of VESC’, the categories refer to increasing severity or size of pathology with ‘Category 1’ being the least severe. For ‘herniations’, the categories represent the following types: 1 = focal protrusion; 2 = broad-based protrusion; 3 = extrusion; 4 = sequestration. For ‘type of VESC’, the categories refer to the following types: 1 = Type I; 2 = Type II; 3 = Type III; 4 = mixed Type I/II; 5 = mixed Type II/III; 6 = mixed Type I/III. For ‘anterolisthesis’ the categories refer to the Meyerdings classification. Detailed information on proportions of categories of MRI findings is described in Additional file 2. **(b)** The mean age of the motion segments was 48 years (SD 9) and the proportion of women was 51%. **(c)** VESC = vertebral endplate signal change; Cat. = categories; EP = endplate.

**Figure 6:**
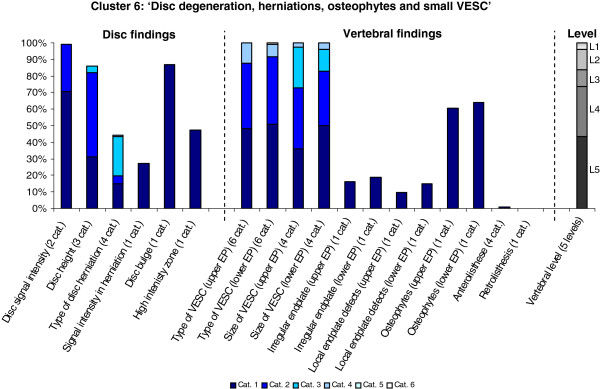
**Proportion of categories of MRI findings for Cluster 6 containing 3.3% of the 3155 motion-segments. (a)** The vertical bars on the graph represent the proportion of vertebral motion segments present within each coding category for each MRI finding. The bars on the graph do not show the ‘Category 0’ as that represents the presence of only normal findings. For pathologies with only one category, it represents the presence of the pathology. For ‘disc signal intensity’, ‘disc height’ and ‘size of VESC’, the categories refer to increasing severity or size of pathology with ‘Category 1’ being the least severe. For ‘herniations’, the categories represent the following types: 1 = focal protrusion; 2 = broad-based protrusion; 3 = extrusion; 4 = sequestration. For ‘type of VESC’, the categories refer to the following types: 1 = Type I; 2 = Type II; 3 = Type III; 4 = mixed Type I/II; 5 = mixed Type II/III; 6 = mixed Type I/III. For ‘anterolisthesis’, the categories refer to the Meyerdings classification. Detailed information on proportions of categories of MRI findings is described in Additional file 2. **(b)** The mean age of the motion segments was 47 years (SD 9) and the proportion of women was 53%. **(c)** VESC = vertebral endplate signal change; Cat. = categories; EP = endplate.

**Figure 7:**
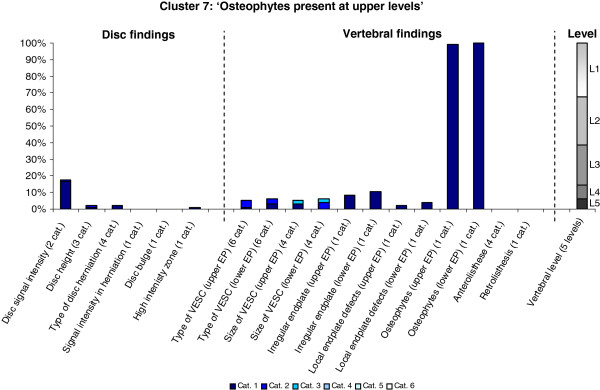
**Proportion of categories of MRI findings for Cluster 7 containing 3.0% of the 3155 motion-segments. (a)** The vertical bars on the graph represent the proportion of vertebral motion segments present within each coding category for each MRI finding. The bars on the graph do not show the ‘Category 0’, as that represents the presence of only normal findings. For pathologies with only one category, it represents the presence of the pathology. For ‘disc signal intensity’, ‘disc height’ and ‘size of VESC’, the categories refer to increasing severity or size of pathology with ‘Category 1’ being the least severe. For ‘herniations’, the categories represent the following types: 1 = focal protrusion; 2 = broad-based protrusion; 3 = extrusion; 4 = sequestration. For ‘type of VESC’, the categories refer to the following types: 1 = Type I; 2 = Type II; 3 = Type III; 4 = mixed Type I/II; 5 = mixed Type II/III; 6 = mixed Type I/III. For ‘anterolisthesis’, the categories refer to the Meyerdings classification. Detailed information on proportions of categories of MRI findings is described in Additional file 2. **(b)** The mean age of the motion segments was 45 years (SD 9) and the proportion of women was 64%. **(c)** VESC = vertebral endplate signal change; Cat. = categories; EP = endplate.

**Figure 8:**
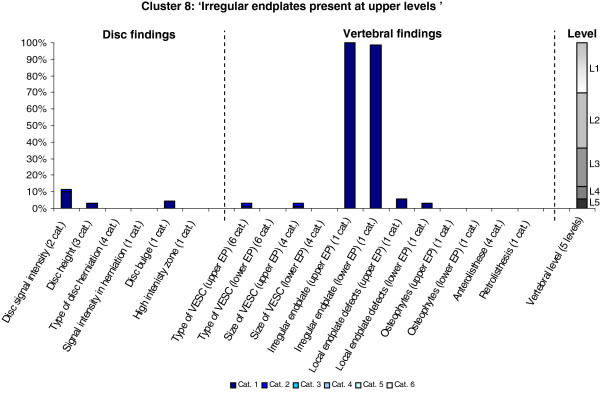
**Proportion of categories of MRI findings for Cluster 8 containing 2.1% of the 3155 motion-segments. (a)** The vertical bars on the graph represent the proportion of vertebral motion segments present within each coding category for each MRI finding. The bars on the graph do not show the ‘Category 0’ as that represents the presence of only normal findings. For pathologies with only one category, it represents the presence of the pathology. For ‘disc signal intensity’, ‘disc height’ and ‘size of VESC’, the categories refer to increasing severity or size of pathology with ‘Category 1’ being the least severe. For ‘herniations’, the categories represent the following types: 1 = focal protrusion; 2 = broad-based protrusion; 3 = extrusion; 4 = sequestration. For ‘type of VESC’, the categories refer to the following types: 1 = Type I; 2 = Type II; 3 = Type III; 4 = mixed Type I/II; 5 = mixed Type II/III; 6 = mixed Type I/III. For ‘anterolisthesis’, the categories refer to the Meyerdings classification. Detailed information on proportions of categories of MRI findings is described in Additional file 2. **(b)** The mean age of the motion segments was 35 years (SD 9) and the proportion of women was 28%. **(c)** VESC = vertebral endplate signal change; Cat. = categories; EP = endplate.

**Figure 9:**
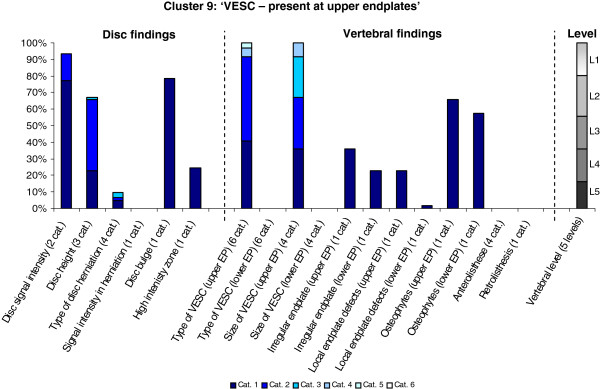
**Proportion of categories of MRI findings for Cluster 9 containing 2.0% of the 3155 motion-segments. (a)** The vertical bars on the graph represent the proportion of vertebral motion segments present within each coding category for each MRI finding. The bars on the graph do not show the ‘Category 0’, as that represents the presence of only normal findings. For pathologies with only one category it represents the presence of the pathology. For ‘disc signal intensity’, ‘disc height’ and ‘size of VESC’, the categories refer to increasing severity or size of pathology with ‘Category 1’ being the least severe. For ‘herniations’, the categories represent the following types: 1 = focal protrusion; 2 = broad-based protrusion; 3 = extrusion; 4 = sequestration. For ‘type of VESC’, the categories refer to the following types: 1 = Type I; 2 = Type II; 3 = Type III; 4 = mixed Type I/II; 5 = mixed Type II/III; 6 = mixed Type I/III. For ‘anterolisthesis’, the categories refer to the Meyerdings classification. Detailed information on proportions of categories of MRI findings is described in Additional file 2. **(b)** The mean age of the motion segments was 47 years (SD 7) and the proportion of women was 34%. **(c)** VESC = vertebral endplate signal change; Cat. = categories; EP = endplate.

**Figure 10:**
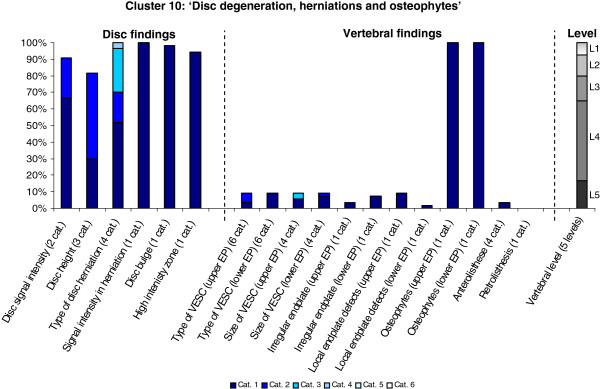
**Proportion of categories of MRI findings for Cluster 10 containing 1.7% of the 3155 motion-segments. (a)** The vertical bars on the graph represent the proportion of vertebral motion segments present within each coding category for each MRI finding. The bars on the graph do not show the ‘Category 0’, as that represents the presence of only normal findings. For pathologies with only one category it represents the presence of the pathology. For ‘disc signal intensity’, ‘disc height’ and ‘size of VESC’, the categories refer to increasing severity or size of pathology with ‘Category 1’ being the least severe. For ‘herniations’, the categories represent the following types: 1 = focal protrusion; 2 = broad-based protrusion; 3 = extrusion; 4 = sequestration. For ‘type of VESC’, the categories refer to the following types: 1 = Type I; 2 = Type II; 3 = Type III; 4 = mixed Type I/II; 5 = mixed Type II/III; 6 = mixed Type I/III. For ‘anterolisthesis’, the categories refer to the Meyerdings classification. Detailed information on proportions of categories of MRI findings is described in Additional file 2. **(b)** The mean age of the motion segments was 43 years (SD 10) and the proportion of women was 50%. **(c)** VESC = vertebral endplate signal change; Cat. = categories; EP = endplate.

**Figure 11:**
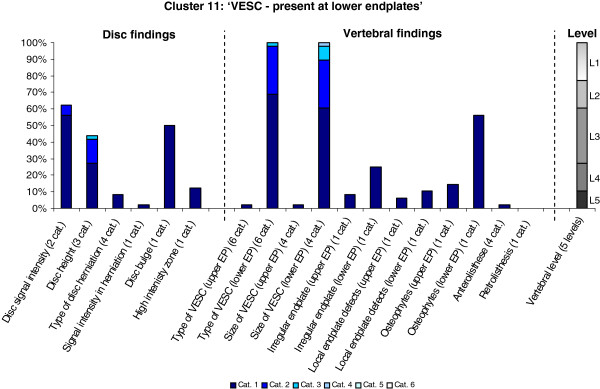
**Proportion of categories of MRI findings for Cluster 11 containing 1.6% of the 3155 motion-segments. (a)** The vertical bars on the graph represent the proportion of vertebral motion segments present within each coding category for each MRI finding. The bars on the graph do not show the ‘Category 0’, as that represents the presence of only normal findings. For pathologies with only one category, it represents the presence of the pathology. For ‘disc signal intensity’, ‘disc height’ and ‘size of VESC’, the categories refer to increasing severity or size of pathology with ‘Category 1’ being the least severe. For ‘herniations’, the categories represent the following types: 1 = focal protrusion; 2 = broad-based protrusion; 3 = extrusion; 4 = sequestration. For ‘type of VESC’, the categories refer to the following types: 1 = Type I; 2 = Type II; 3 = Type III; 4 = mixed Type I/II; 5 = mixed Type II/III; 6 = mixed Type I/III. For ‘anterolisthesis’, the categories refer to the Meyerdings classification. Detailed information on proportions of categories of MRI findings is described in Additional file 2. **(b)** The mean age of the motion segments was 44 years (SD 10) and the proportion of women was 42%. **(c)** VESC = vertebral endplate signal change; Cat. = categories; EP = endplate.

**Figure 12:**
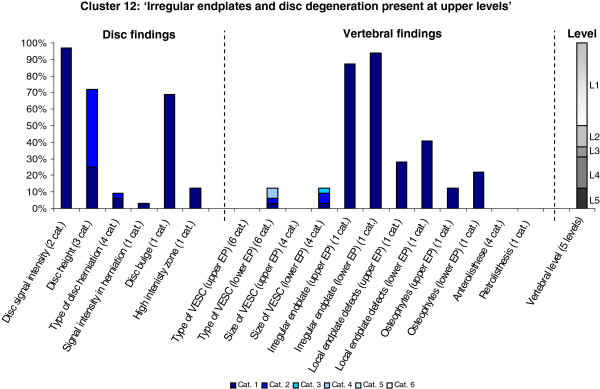
**Proportion of categories of MRI findings for Cluster 12 containing 1.1% of the 3155 motion-segments. (a)** The vertical bars on the graph represent the proportion of vertebral motion segments present within each coding category for each MRI finding. The bars on the graph do not show the ‘Category 0’, as that represents the presence of only normal findings. For pathologies with only one category, it represents the presence of the pathology. For ‘disc signal intensity’, ‘disc height’ and ‘size of VESC’, the categories refer to increasing severity or size of pathology with ‘Category 1’ being the least severe. For ‘herniations’, the categories represent the following types: 1 = focal protrusion; 2 = broad-based protrusion; 3 = extrusion; 4 = sequestration. For ‘type of VESC’ the categories refer to the following types: 1 = Type I; 2 = Type II; 3 = Type III; 4 = mixed Type I/II; 5 = mixed Type II/III; 6 = mixed Type I/III. For ‘anterolisthesis’ the categories refer to the Meyerdings classification. Detailed information on proportions of categories of MRI findings is described in Additional file 2. **(b)** The mean age of the motion segments was 37 years (SD 12) and the proportion of women was 41%. **(c)** VESC = vertebral endplate signal change; Cat. = categories; EP = endplate.

The Latent Class Analysis identified twelve clusters of MRI findings. One cluster, characterised by no abnormal MRI findings, contained 52% of the 3,155 motion segments and represented the normal, pre-degenerative state. The second largest cluster contained almost 15% of the motion segments and was characterised by motion segments with reduced disc height, reduced disc signal intensity, disc bulges and a minor degree of disc protrusions and high intensity zones. Sixty percent of these changes were located at the two lowest spinal levels. The rest of the clusters were smaller, with each containing less than 8% of the motion segments. Each cluster and its distribution of MRI findings is shown diagrammatically in Figures 1, 2, 3, 4, 5, 6, 7, 8, 9, 10, 11, and 12. More detailed descriptive data of the MRI findings at a cluster level are tabulated in Additional file 2.

### Hypothetical pathoanatomic pathways

**Figure 13:**
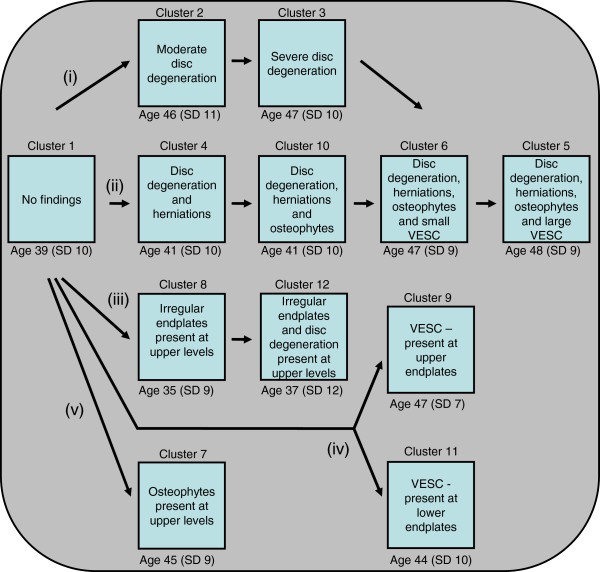
**Degenerative pathways of the disco-vertebral complex.** ‘Age’ is the average chronological age of the vertebral segments in each cluster, ‘SD’ is the standard deviation and ‘VESC’ is vertebral endplate signal change. The clusters were divided into the following five pathways: **(i)** Progressive stages of disc degeneration in the lower lumbar levels. **(ii)** Progressive stages of disc herniations and VESC in the lower lumbar levels. **(iii)** Progressive endplate changes and disc degeneration at the upper lumbar levels only. **(iv)** VESC only at either the upper or the lower endplates. **(v)** Osteophytes at the upper lumbar motion segments.

The hypothetical pathoanatomic pathways derived from the content analysis of the clusters were: (i) two clusters representing progressive stages of disc degeneration in the lower lumbar levels; (ii) four clusters representing progressive stages of disc herniations and VESC in the lower lumbar levels; (iii) two clusters containing progressive endplate changes and disc degeneration at the upper lumbar levels only; (iv) two cluster with VESC only, one cluster each for changes in the upper and the lower endplates; and lastly, (v) one cluster containing osteophytes at the upper lumbar motion segments. The pathoanatomic pathways and their clusters are illustrated in Figure 13.

Once the pathoanatomic pathways had been categorised, the mean age of the motion segments in each cluster was added to Figure 13 as a post-hoc analysis. The average age of the vertebral segments within each cluster supported the notion of a developmental model, as the chronological age in each cluster consistently increased across each pathoanatomic pathway as degeneration developed. Although the chronological age and biological age of vertebral segments may not be the same due to pathological degeneration, the observed age progression across the biological pathways adds credibility to the construct validity of this classification.

## Discussion

In this study, comprehensive MRI findings of lumbar vertebral motion segments were grouped using Latent Class Analysis into twelve clusters. Using content analysis, the identified clusters were then qualitatively grouped into five different biological pathways of degeneration that appear to have face validity.

This data-driven approach of the analysis of MRI findings is novel in LBP and our proof-of-concept study shows that this methodological approach produces plausible results suitable for further investigation. Two previous studies on LBP have used a multivariable approach to exploring the relationship between MRI findings and clinical variables, but in contrast to the current study, their multivariable analysis clustered the clinical variables and related these to single MRI findings. Takatalo et al. [27,28] used Latent Class Analyses to group multiple clinical variables (including pain, activity limitation and care seeking) from 468 people into five clusters which were then investigated for their association with individual MRI findings. It is not yet known whether the most revealing investigations of the association between MRI findings and clinical characteristics will involve the clustering of findings from both domains, only one domain or neither domain. In other areas of research, such as neurology and biological psychiatry, Latent Class Analysis has been used with MRI findings to investigate the validity of diagnostic subgroups or the accuracy of diagnostic tests [29-32].

Although the emphasis in the current study was on method, the identified MRI clusters appear to be biologically plausible and could be grouped into pathways of degeneration. These suggested pathways were based on the available evidence about the histological progression of disco-vertebral degeneration for different age groups [13] and on classification systems of degeneration based on histological [33] and MRI findings [19]. The chronology of the degenerative pathways was also informed by knowledge of the potential reversibility of some MRI findings (for example, an intervertebral disc herniation is potentially reversible but reduced disc height is not) and also, if the distribution of findings were mainly located in the lower or the upper lumbar motion segments. However, as there are few studies describing the precise association between the histology of degeneration and specific MRI findings, the evidence on which to base such grouping of MRI clusters is incomplete. Therefore, the clusters and degenerative pathways should be interpreted with caution and viewed as hypothesis-setting only.

MRIs of the lumbar spine can provide researchers with highly detailed information on spinal pathoanatomy, especially when quantified via comprehensive research protocols [21,22]. However, the volume of this detail can present a challenge for data analysis. For example, in our study, for each disco-vertebral level, there were more than 50 variables, each with up to seven response options. Traditional approaches to data reduction, such as regression analysis, can model the dominant pattern of association between MRI variables but do not accommodate the presence of multiple patterns that may reflect different stages of degeneration or different pathological pathways. Clustering techniques, such as Latent Class Analysis, have the advantage of allowing for that complexity.

We interpret these results as indicating that this research method should be further tested in different datasets to investigate if similar clusters are consistently replicated or if such clusters are highly sample-specific. If these clusters are reproducible, they could provide a platform from which to investigate associations between multivariate MRI findings and clinical findings, such as pain, activity limitations or other clinically relevant outcomes. Similarly, a further extension of this work would be to include MRI findings of structures other than the disco-vertebral complex, such as MRI findings of the vertebral canal, zygapophyseal joints and para-spinal soft tissue structures.

The strengths of this study are that a new statistical approach was applied to a large sample of MRI findings that had been rigorously described by an experienced research radiologist using a data extraction protocol with high reproducibility, and the results were cautiously interpreted. A weakness of the study is that its methodological focus means that the content of the identified clusters and biological pathways are preliminary and require extensive further study before their clinical validity, if any, can be determined.

## Conclusions

This study has shown that Latent Class Analysis can be used to identify clusters of MRI findings from people with LBP and that those clusters can be grouped into biological pathways that have some face validity. If, when applied to other datasets of similar patients, this research method identifies reproducible clusters of MRI findings, these may form a stable platform to investigate the relationship between degenerative pathways and important clinical characteristics such as pain and activity limitation.

## Abbreviations

LBP: Low back pain; MRI: Magnetic resonance imaging; SD: Standard deviation; VESC: Vertebral endplate signal change.

## Competing interests

The authors declare that they have no competing interests.

## Authors’ contributions

RKJ, TSJ, PK and PMK all participated in conception and design of the project and in interpretation of the data. RKJ and PMK performed the analyses and wrote the draft manuscript. All authors have made substantial contributions to revising the manuscript. All authors read and approved the final manuscript.

## Pre-publication history

The pre-publication history for this paper can be accessed here:

http://www.biomedcentral.com/1471-2474/14/198/prepub

## Supplementary Material

Additional file 1 **MRI findings and their distribution in the whole sample of vertebral segments.** Detailed descriptive data of the MRI findings at a whole group level are tabulated in Additional file 1. Click here for file

Additional file 2 **The distribution (%) of the MRI findings in the 12 clusters.** Detailed descriptive data of the MRI findings at a cluster level are tabulated in Additional file 2. Click here for file
