# Supplementary material for: Can pathoanatomical pathways of degeneration in lumbar motion segments be identified by clustering MRI findings
Source: BMC Musculoskelet Disord. 2013 Jul 1;14:198. doi: 10.1186/1471-2474-14-198 (PMC3706235; doi:10.1186/1471-2474-14-198)
Supplement: Additional file 1 — MRI findings and their distribution in the whole sample of vertebral segments. Detailed descriptive data of the MRI findings at a whole group level are tabulated in Additional file 1. [file 1471-2474-14-198-S1.pdf]

Additional file 1 - MRI findings and their distribution in the whole sample of vertebral segments.

	N	%
**Disc n=3153***		
**Disc signal intensity**		
Hyperintense with visible intranuclear cleft	1,711	54
Intermediate signal intensity	1,165	37
Hypointense	277	9
**Disc height**		
Disc higher than the disc above	2,026	64
Disc as high as the disc above (if normal)	453	14
Disc narrower than the disc above (if normal)	626	20
Endplates almost in contact	48	2
**Type of protrusion**		
No protrusion	2,681	85
Focal protrusion	211	7
Broad-based protrusion	70	2
Extrusion	185	6
Sequestration	6	0.2
**Signal intensity in protrusion**		
No	2,848	90
Yes	305	10
**Disc bulge**		
No	1,911	61
Yes	1,242	39
**High intensity zone**		
No	2,574	82
Yes	579	18
**Vertebra n=3155**		
**Type of VESC (upper endplate)**		
None	2,793	89
Type I	153	5
Type II	140	4
Type III	1	0.03
Mixed I/II	40	1
Mixed II/III	15	0.5
Mixed I/III	13	0.4
**Type of VESC (lower endplate)**		
None	2,790	88
Type I	180	6
Type II	125	4
Type III	2	0.1
Mixed I/II	33	1
Mixed II/III	12	0.4
Mixed I/III	13	0.4
**Size of VESC (upper endplate)**		
None	2,793	89
Endplate only	90	3
<25%	104	3
25-50%	99	3

>50%	69	2
**Size of VESC (lower endplate)**		
None	2,790	88
Endplate only	127	4
<25%	125	4
25-50%	85	3
>50%	28	1
**Irregular endplate (upper endplate)**		
No	2,736	87
Yes	419	13
**Irregular endplate (lower endplate)**		
No	2,720	86
Yes	435	14
**Local endplate defects (upper endplate)**		
No	3,030	96
Yes	125	4
**Local endplate defects (lower endplate)**		
No	3,049	97
Yes	106	3
**Osteophytes (upper endplate)**		
No	2,484	79
Yes	671	21
**Osteophytes (lower endplate)**		
No	2,446	78
Yes	709	22
**Anterolisthesis**		
Normal	3,118	99
Meyerding grade 1	36	1
Meyerding grade 2	1	0.03
Meyerding grade 3	0	0
Meyerding grade 4	0	0
**Retrolisthesis**		
No	3,148	99.8
Yes	7	0.2

* MRI information for two discs in one person was unavailable due to previous fusion surgery and the resultant absence of these discs.

VESC: Vertebral endplate signal change
