# Supplementary material for: Can pathoanatomical pathways of degeneration in lumbar motion segments be identified by clustering MRI findings
Source: BMC Musculoskelet Disord. 2013 Jul 1;14:198. doi: 10.1186/1471-2474-14-198 (PMC3706235; doi:10.1186/1471-2474-14-198)
Supplement: Additional file 2 — The distribution (%) of the MRI findings in the 12 clusters. Detailed descriptive data of the MRI findings at a cluster level are tabulated in Additional file 2. [file 1471-2474-14-198-S2.pdf]

Additional file 2 - The distribution (%) of the MRI findings in the 12 clusters

	Category	Cluster 1	Cluster 2	Cluster 3	Cluster 4	Cluster 5	Cluster 6	Cluster 7	Cluster 8	Cluster 9	Cluster 10	Cluster 11	Cluster 12
**Disc n=3153***													
**Disc signal intensity**													
Hyperintense with visible intranuclear cleft	0	91.9	5.3	2.9	4.5	98.8	.9	82.3	88.4	6.6	9.3	98.8	3.1
Intermediate signal intensity	1	8.1	77.4	77.8	76.7	.8	70.8	16.7	10.1	77.0	66.7	.8	96.9
Hypointense	2	0	17.3	19.3	18.8	.4	28.3	1.0	1.4	16.4	24.1	.4	0
**Disc height**													
Disc higher than the disc above	0	98.8	22.2	10.7	19.3	5.8	14.2	97.9	97.1	32.8	18.5	5.8	28.1
Disc as high as the disc above (if normal)	1	.8	41.7	32.1	36.9	8.8	31.1	1.0	2.9	23.0	29.6	8.8	25.0
Disc narrower than the disc above (if normal)	2	.4	35.7	55.6	43.2	64.9	50.9	1.0	0	42.6	51.9	64.9	46.9
endplates almost in contact	3	0	.4	1.6	.6	20.5	3.8	0	0	1.6	0	20.5	0
**Type of protrusion**													
No protrusion	0	99.8	83.5	90.1	0	56.1	55.7	97.9	100	90.2	0	56.1	90.6
Focal protrusion	1	.2	8.5	4.5	47.7	10.5	15.1	2.1	0	4.9	51.9	10.5	6.3
Broad-based protrusion	2	0	1.7	1.2	11.9	12.3	4.7	0	0	1.6	18.5	12.3	3.1
Extrusion	3	.1	6.2	4.1	38.6	21.1	23.6	0	0	3.3	25.9	21.1	0
Sequestration	4	0	0	0	1.7	0	.9	0	0	0	3.7	0	0
**Signal intensity in protrusion**													
No	0	100	100	100	0	74.3	72.6	100	100	100	0	74.3	96.9
Yes	1	0	0	0	100	25.7	27.4	0	0	0	100	25.7	3.1
**Disc bulge**													
No	0	98.5	13.0	4.1	4.5	2.3	13.2	100	95.7	21.3	1.9	2.3	31.3
Yes	1	1.5	87.0	95.9	95.5	97.7	86.8	0	4.3	78.7	98.1	97.7	68.8
**High intensity zone**													
No	0	99.9	67.3	75.7	6.8	56.7	52.8	99.0	100	75.4	5.6	56.7	87.5
Yes	1	.1	32.7	24.3	93.2	43.3	47.2	1.0	0	24.6	94.4	43.3	12.5
**Vertebra n=3155**													
**Type of VESC (upper EP)**													
None	0	99.9	100	100	94.3	0	0	94.8	97.1	0	90.7	0	100
Type I	1	.1	0	0	2.3	39.2	48.1	1.0	1.4	41.0	3.7	39.2	0
Type II	2	0	0	0	2.8	31.6	39.6	4.2	1.4	50.8	5.6	31.6	0
Type III	3	0	0	0	0	.6	0	0	0	0	0	.6	0
Mixed I/II	4	0	0	0	0	14.0	12.3	0	0	4.9	0	14.0	0
Mixed II/III	5	0	0	0	.6	7.0	0	0	0	3.3	0	7.0	0
Mixed I/III	6	0	0	0	0	7.6	0	0	0	0	0	7.6	0
**Type of VESC (lower EP)**													
None	0	100	100	91.4	97.7	0	0	93.8	100	100	90.7	0	87.5
Type I	1	0	0	4.9	1.7	40.4	50.9	3.1	0	0	9.3	40.4	3.1
Type II	2	0	0	3.7	0	32.2	40.6	3.1	0	0	0	32.2	3.1
Type III	3	0	0	0	0	.6	0	0	0	0	0	.6	0
Mixed I/II	4	0	0	0	0	13.5	7.5	0	0	0	0	13.5	6.3
Mixed II/III	5	0	0	0	.6	6.4	0	0	0	0	0	6.4	0
Mixed I/III	6	0	0	0	0	7.0	.9	0	0	0	0	7.0	0

**Size of VESC (upper EP)**													
None	0	99.9	100	100	94.3	0	0	94.8	97.1	0	90.7	0	100
Endplate only	1	0	0	0	1.7	11.1	35.8	3.1	1.4	36.1	5.6	11.1	0
<25%	2	.1	0	0	2.3	23.4	36.8	0	1.4	31.1	0	23.4	0
25-50%	3	0	0	0	1.7	29.8	24.5	2.1	0	24.6	3.7	29.8	0
>50%	4	0	0	0	0	35.7	2.8	0	0	8.2	0	35.7	0
**Size of VESC (lower EP)**													
None	0	100	100	91.4	97.7	0	0	93.8	100	100	90.7	0	87.5
Endplate only	1	0	0	3.7	.6	17.5	50.0	0	0	0	7.4	17.5	3.1
<25%	2	0	0	2.5	1.7	35.1	33.0	4.2	0	0	1.9	35.1	6.3
25-50%	3	0	0	1.6	0	35.1	13.2	2.1	0	0	0	35.1	3.1
>50%	4	0	0	.8	0	12.3	3.8	0	0	0	0	12.3	0
**Irregular endplate (upper EP)**													
No	0	98.4	97.6	81.5	90.9	0	84.0	91.7	0	63.9	96.3	0	12.5
Yes	1	1.6	2.4	18.5	9.1	100	16.0	8.3	100	36.1	3.7	100	87.5
**Irregular endplate (lower EP)**													
No	0	98.6	95.7	78.6	93.8	0	81.1	89.6	1.4	77.0	92.6	0	6.3
Yes	1	1.4	4.3	21.4	6.3	100	18.9	10.4	98.6	23.0	7.4	100	93.8
**Local endplate defects (upper EP)**													
No	0	98.8	96.8	95.9	96.0	84.8	90.6	97.9	94.2	77.0	90.7	84.8	71.9
Yes	1	1.2	3.2	4.1	4.0	15.2	9.4	2.1	5.8	23.0	9.3	15.2	28.1
**Local endplate defects (lower EP)**													
No	0	98.6	96.4	94.2	98.3	95.9	84.9	95.8	97.1	98.4	98.1	95.9	59.4
Yes	1	1.4	3.6	5.8	1.7	4.1	15.1	4.2	2.9	1.6	1.9	4.1	40.6
**Osteophytes (upper EP)**													
No	0	99.9	98.5	.4	98.9	9.9	39.6	1.0	100	34.4	0	9.9	87.5
Yes	1	.1	1.5	99.6	1.1	90.1	60.4	99.0	0	65.6	100	90.1	12.5
**Osteophytes (lower EP)**													
No	0	99.7	95.5	0	99.4	11.1	35.8	0	100	42.6	0	11.1	78.1
Yes	1	.3	4.5	100	.6	88.9	64.2	100	0	57.4	100	88.9	21.9
**Anterolistese**													
Normal	0	99.9	97.6	97.1	99.4	93.0	99.1	100	100	100	96.3	93.0	100
Meyerding grade 1	1	.1	2.1	2.9	.6	7.0	.9	0	0	0	3.7	7.0	0
Meyerding grade 2	2	0	.2	0	0	0	0	0	0	0	0	0	0
Meyerding grade 3	3	0	0	0	0	0	0	0	0	0	0	0	0
Meyerding grade 4	4	0	0	0	0	0	0	0	0	0	0	0	0
**Retrolisthesis**													
No	0	100	99.8	100	100	96.5	100	100	100	100	100	96.5	100
Yes	1	0	.2	0	0	3.5	0	0	0	0	0	3.5	0
**Motion segment n=3155**													
L1-L2	L1	13.7	10.0	18.5	1.7	53.2	3.7	32.3	30.4	19.7	7.4	53.2	50.0
L2-L3	L2	12.7	10.3	23.5	4.0	29.8	12.3	29.1	33.3	24.6	13.0	29.8	12.5
L3-L4	L3	22.0	18.2	27.1	12.0	7.0	10.4	24.0	23.2	19.7	14.8	7.0	6.3
L4-L5	L4	25.0	31.6	24.3	39.2	7.6	30.2	8.3	7.3	19.7	48.1	7.6	18.7
L5-S1	L5	26.6	29.9	6.6	43.1	2.4	43.4	6.3	5.8	16.3	16.7	2.4	12.5

* MRI information for two discs in one person was unavailable due to previous fusion surgery and the resultant absence of these discs.

VESC = Vertebral endplate signal change; EP = endplate
